# Earthquake Early Warning System for Izmir, Western Anatolia, Türkiye Based on Multi-Station Similarity Analysis and Real-Time Seismic Data Processing

**DOI:** 10.3390/s26102931

**Published:** 2026-05-07

**Authors:** Yunus Doğan, Ahmet Başbuğ, Fatih Semirgin, Yusuf Eren Kaya, Orkun Çınar, Hasan Sözbilir, Özkan Cevdet Özdağ, Reyat Yılmaz, Alp Kut, Özgür Tamer, Recep Çakır, Mehmet Utku, Özgür Özçelik, Mustafa Softa

**Affiliations:** 1 Department of Computer Engineering, Dokuz Eylül University, Izmir 35390, Türkiye; ahmet.basbug@ogr.deu.edu.tr (A.B.); fatih.semirgin@ogr.deu.edu.tr (F.S.); yusuf.kaya@ogr.deu.edu.tr (Y.E.K.); orkun@cs.deu.edu.tr (O.Ç.); alp@cs.deu.edu.tr (A.K.); 2 Department of Geological Engineering, Dokuz Eylül University, Izmir 35390, Türkiye; hasan.sozbilir@deu.edu.tr (H.S.); mustafa.softa@deu.edu.tr (M.S.); 3 Earthquake Research and Application Center, Dokuz Eylül University, Izmir 35390, Türkiye; 4 Department of Geophysical Engineering, Dokuz Eylül University, Izmir 35390, Türkiye; cevdet.ozdag@deu.edu.tr (Ö.C.Ö.); mehmet.utku@deu.edu.tr (M.U.); 5 Department of Electrical and Electronics Engineering, Dokuz Eylül University, Izmir 35390, Türkiye; reyad.yilmaz@deu.edu.tr (R.Y.); ozgur.tamer@deu.edu.tr (Ö.T.); 6 Civil Engineering Department, İzmir Institute of Technology, Izmir 35430, Türkiye; recep.cakir@dnr.wa.gov; 7 Civil Engineering Department, Dokuz Eylül University, Izmir 35390, Türkiye; ozgur.ozcelik@deu.edu.tr

**Keywords:** earthquake early warning system, multi-station similarity analysis, real-time seismic detection, artificial intelligence, seismic signal processing

## Abstract

Earthquake Early Warning Systems (EEWS) represent one of the most effective technological solutions for mitigating the impacts of strong ground motion in seismically active regions. This study presents the design, implementation, and comprehensive evaluation of a real-time earthquake early warning system for Izmir-a region in Western Anatolia characterized by complex tectonic structures and high seismic hazard-using multi-station seismic acceleration data. The proposed framework integrates multi-threaded data acquisition, signal preprocessing, Min-Max normalization, and Euclidean distance-based similarity analysis to enable rapid detection of anomalous seismic patterns during the early P-wave phase. The system architecture consists of distributed sensor inputs, centralized real-time processing, similarity-based anomaly detection, and user-oriented visualization and alerting modules. The performance of the system was evaluated using both real and synthetic seismic datasets. Instrumental earthquake catalog from the 12 June 2017 Karaburun (Mw 6.2) and 30 October 2020 Samos (Mw 6.6) earthquakes demonstrate that the system can generate early warnings 18 s and 13 s prior to strong ground shaking, respectively. In addition, synthetic seismic scenarios were employed to assess system robustness under varying noise levels, station configurations, and signal conditions. The results indicate that the proposed framework maintains stable detection performance and low false-positive rates across diverse operational scenarios. The methodology emphasizes computational efficiency and inter-station waveform coherence analysis, providing a lightweight alternative to conventional magnitude-based approaches. By avoiding computationally intensive source inversion, the system achieves low-latency performance while preserving detection reliability. The proposed EEWS demonstrates strong generalization capability, scalability, and practical applicability for real-time deployment in earthquake-prone urban environments.

## 1. Introduction

Earthquakes are among the most destructive natural hazards due to their sudden occurrence and rapid energy release. In tectonically active regions, they can cause severe structural damage, economic losses, and casualties. Türkiye is located within one of the most seismically active regions of the world, influenced by the interaction of major tectonic plates. In particular, the western Anatolian region exhibits active fault systems, and Izmir is highly vulnerable to seismic hazards, with historical evidence indicating the potential for earthquakes exceeding magnitude 7.0 [1]. The 30 October 2020 Mw 6.6 Samos earthquake further demonstrated the need for effective early warning mechanisms.

Although deterministic earthquake prediction remains infeasible, Earthquake Early Warning Systems (EEWSs) provide a practical solution by exploiting the velocity difference between P-waves and S-waves. By detecting the initial P-wave motion, EEWSs can issue alerts seconds before strong ground shaking occurs, enabling automated safety measures and reducing risk [2]. Consequently, the development of efficient and reliable EEWSs has become an important research focus.

Existing EEWS approaches have evolved significantly, including threshold-based methods, Bayesian frameworks, waveform-based models, and large-scale operational systems. Threshold-based systems offer fast detection but are sensitive to noise and station density [3]. Bayesian approaches such as the Virtual Seismologist improve parameter estimation but increase computational complexity [4]. Large-scale numerical and parallel computing methods enhance seismic modeling but are often unsuitable for low-latency applications [5]. Operational systems such as ShakeAlert highlight trade-offs between detection speed and accuracy [6,7].

Alternative sensing strategies, including smartphone-based seismic networks, offer scalability but introduce variability in sensor quality and synchronization [8,9]. Reviews indicate that modern EEWS research increasingly integrates distributed sensing, mobile platforms, and artificial intelligence [10]. Network-based solutions emphasize communication reliability and system topology [11], while national-scale systems demonstrate both effectiveness and limitations, including magnitude underestimation and computational demands [12,13]. Communication infrastructure and algorithmic reliability also remain critical factors in system performance [14,15].

From a signal processing perspective, waveform similarity and spectral characteristics provide important theoretical foundations for seismic detection [16], while geospatial calculations such as the Haversine method are widely used in network analysis and are essential in this study for computing distances between seismic stations and defining spatial relationships in the multi-station detection framework [17]. Regional implementations such as PRESTo, Taiwan EEWS, and Indian systems illustrate varying methodological approaches depending on seismic conditions [18,19,20]. In Türkiye, studies such as IERREWS and smartphone-based detection systems have contributed to regional early warning development [8,21,22].

In addition to the aforementioned approaches, recent advancements have further expanded EEWS capabilities. The APPLES algorithm improves ground-motion prediction and reduces false alerts under certain conditions [23]. Deep learning-based approaches enable rapid magnitude estimation with improved accuracy, although they require large datasets and computational resources [24]. Regional magnitude scaling relationships enhance system reliability through local calibration [25]. Infrastructure-oriented implementations, such as railway-specific EEWSs and lightweight server architectures, demonstrate practical deployment strategies [26,27]. Studies on public response highlight the importance of minimizing false alarms to maintain trust [28], while multi-algorithm platforms improve detection stability [29]. Broader risk assessment approaches and machine learning applications further extend EEWS capabilities in hazard mitigation and damage prediction [30,31,32]. Despite these advancements, several challenges persist, including computational complexity, noise sensitivity, infrastructure dependency, latency–accuracy trade-offs, and limited adaptability to regional-scale systems. Most existing approaches rely on magnitude estimation or complex inversion, which may delay alert generation.

Unlike many existing EEWS frameworks that rely on magnitude inversion, probabilistic modeling, or multi-algorithm pipelines, often introducing processing delays on the order of several hundred milliseconds to seconds, the proposed system is designed as a lightweight, real-time framework with a computational latency of approximately 50 ms. This reduction minimizes algorithmic delay, allowing warning performance to be primarily constrained by seismic wave propagation rather than processing overhead.

To address these limitations, this study proposes a computationally efficient, multi-station similarity-based EEWS tailored for Izmir. The system focuses on early-stage waveform coherence rather than magnitude estimation, utilizing normalized signal vectors and Euclidean similarity metrics for rapid detection. By integrating geophysical principles with multi-threaded real-time processing, the proposed framework aims to achieve a balance between detection speed, robustness, and scalability.

The system is evaluated using real earthquake datasets, including the 2017 Karaburun and 2020 Samos events, demonstrating measurable early warning lead times under realistic conditions. The proposed architecture is designed to be adaptable and scalable, providing a practical solution for regional EEWS deployment. These two events were selected because they represent the most significant recent earthquakes affecting the Izmir region, providing high-quality multi-station recordings and diverse seismic wave propagation characteristics. The Karaburun earthquake represents a near-field regional event, while the Samos earthquake provides a broader offshore scenario, enabling evaluation under different source-to-station geometries.

Beyond its technical contribution, this work aims to enhance earthquake preparedness and societal resilience in Izmir. Given the city’s dense population and infrastructure, even a few seconds of early warning can significantly reduce risk.

In summary, this study contributes to earthquake early warning research by

- Developing a multi-station similarity-based real-time detection model;
- Integrating geophysical principles with computational efficiency;
- Validating the approach using real seismic datasets;
- Demonstrating measurable early warning lead times for regional earthquakes.

By combining established seismological concepts with efficient real-time processing, the proposed system provides a practical and regionally adaptable EEWS framework.

The remainder of this paper is organized as follows. Section 1.1 discusses the advantages of the proposed system compared to existing EEWS approaches. Section 2 presents the materials and methodology, including data processing and modeling. Section 3 provides experimental results and performance evaluation. Section 4 discusses the findings, limitations, and future work.

### 1.1. Advantages of the Proposed System Compared to Existing EEWS Approaches

Recent advancements in earthquake early warning systems (EEWSs) have primarily focused on rapid magnitude estimation, machine learning integration, and real-time ground motion prediction. However, these approaches often introduce trade-offs between computational complexity, latency, and robustness [6,9].

A significant research direction involves deep learning- and machine learning-based magnitude estimation, which can improve predictive accuracy but requires large labeled datasets and highly computational resources [33,34,35]. In contrast, the proposed system adopts a model-free, signal-driven approach, eliminating dependency on training data and avoiding challenges related to overfitting and data scarcity.

Recent EEWS frameworks also employ probabilistic and hybrid multi-algorithm architectures, which enhance detection reliability but increase system complexity and latency [6,29]. The proposed method addresses this issue by utilizing a simpler multi-station waveform coherence mechanism, providing a computationally efficient alternative while maintaining robustness.

Ground-motion prediction approaches, such as the APPLES algorithm, further improve intensity estimation but depend on reliable magnitude calculations and additional modeling steps, which may delay alert generation [23]. In contrast, the proposed system bypasses magnitude estimation and focuses on early-stage waveform similarity, enabling faster detection during the P-wave phase.

EEWS performance is also significantly affected by noise, site effects, and sensor variability, particularly in single-station or threshold-based systems [9,34]. The proposed method mitigates these limitations by enforcing multi-station spatial consistency, ensuring that detections are based on coherent seismic wave propagation rather than localized disturbances.

Recent studies have also explored low-cost and IoT-based EEWS architectures, such as smartphone-based seismic networks and lightweight communication systems, which offer scalability but suffer from heterogeneous sensor quality and synchronization challenges [7,27]. The proposed system addresses these limitations through normalization, synchronization, and amplitude-independent similarity analysis.

Latency reduction has been identified as a critical factor in EEWS performance, often more impactful than marginal improvements in magnitude estimation accuracy [36,37]. The proposed system achieves low-latency performance through computationally efficient similarity calculations, parallel processing, and the elimination of inversion-based methods.

Finally, unlike many modern systems optimized for large-scale national infrastructures, the proposed framework is designed for regional and medium-scale deployments, providing a lightweight and scalable solution adaptable to local seismic networks. In summary, the proposed system provides

- Independence from large training datasets;
- Reduced computational complexity;
- Robust detection via multi-station coherence;
- Low-latency performance without magnitude inversion;
- Improved resilience to noise and sensor variability;
- Practical applicability for regional EEWS implementation.

These features position the proposed method as a robust, efficient, and scalable alternative to recent data-intensive EEWS approaches.

## 2. Materials and Methods

### 2.1. Study Area, Seismic Context and Data Source

**Figure 1:**
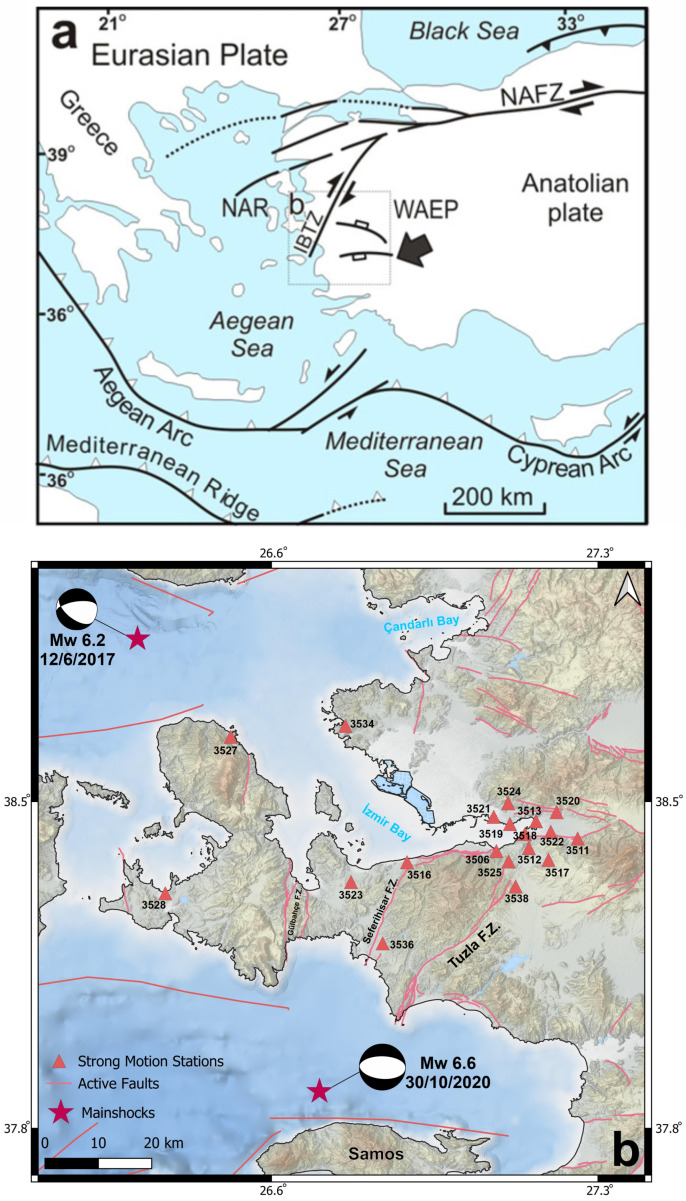
(**a**) Tectonic setting of the Western Anatolia Extensional Province (WAEP). The İzmir-Balıkesir Transfer Zone (IBTZ) defines the boundary between the normal-fault-dominated West Anatolian Extensional Province (WAEP) and the strike-slip-dominated North Aegean Region (NAR). Abbreviations: NAFZ, North Anatolian Fault Zone; AA, Aegean Arc; CA, Cyprian Arc. Modified from Bozkurt (2001) [40] and Özkaymak et al. (2013) [41]. (**b**) Surrounding major fault systems. Compiled from Emre et al. (2018) [42].

The study focuses on the metropolitan region of Izmir, located in Western Anatolia extensional province (WAEP) within the Aegean extensional tectonic regime (Figure 1). Bounded by the North Anatolian Fault Zone from the north and the Hellenic subduction zone (Aegean Arc) from the south, WEAP is considered one of the most seismically active regions in the world [38]. As seen in Figure 1, Izmir and its surroundings are characterized by active normal and strike-slip fault systems associated with crustal thinning and graben formation. Major fault zones surrounding the Izmir Gulf include the Izmir Fault, Tuzla Fault, Gülbahçe Fault, and Seferihisar Fault systems as well as submarine active faults [39]. These structures have produced moderate-to-large magnitude earthquakes historically and represent a significant seismic hazard for densely populated districts.

The seismic data were recorded using Guralp Systems CMG-5TD fully digital triaxial force-feedback accelerometers installed at the stations. The seismic dataset utilized in this study was obtained from AFAD-operated strong motion stations distributed across Izmir province. The network comprises accelerometer-based stations that record three orthogonal components of ground acceleration: East-West (E), North-South (N), and Vertical (U). Each station records acceleration time histories with a sampling interval of 0.01 s (100 Hz sampling frequency), providing a highly temporal resolution suitable for early P-wave detection. Two principal earthquake events were selected for detailed performance evaluation:

- High computational complexity in probabilistic or inversion-based systems;
- Karaburun Earthquake (12 June 2017, Mw 6.2) [38];
- Samos Earthquake (30 October 2020, Mw 6.6) [39].

In addition to these main case studies, supplementary background acceleration data recorded during non-seismic periods were incorporated to evaluate false alarm resistance and system stability under normal environmental vibration conditions. The raw acceleration records were initially stored in text-based formats. These records required synchronization and preprocessing due to minor timestamp offsets among stations. The preprocessing pipeline corrected temporal misalignments and ensured consistent window-based comparison across stations.

The background dataset consists of approximately 2 h of continuous recordings obtained from the same stations during non-seismic periods. The data were selected based on time intervals with no recorded earthquake activity in regional catalogs. These recordings include typical environmental and anthropogenic noise sources, ensuring realistic evaluation of false alarm behavior.

Table 1 summarizes the seismological parameters of the Karaburun (2017) and Samos (2020) earthquakes.

The seismic source of the Karaburun earthquake that occurred on 12.06.2017 is associated with the Mytilene Fault, while the Samos earthquake on 30.10.2020 is attributed to the Samos Fault (Table 1).

The geographical coordinates of the seismic stations and their epicentral distances (defined as the distance between the earthquake epicenter and the station location) for the Samos (2020) and Karaburun (2017) earthquakes are presented in Table 2 and Table 3, respectively. These stations form a regional monitoring network distributed across the Izmir metropolitan area, providing sufficient spatial coverage for real-time waveform analysis.

The variation in epicentral distances, ranging from near-field to more distant stations, allows the system to capture diverse waveform characteristics under different propagation conditions. This spatial diversity is essential for evaluating the robustness of the proposed multi-station similarity-based detection framework.

In addition, representative seismic records obtained from these stations are analyzed to identify P-wave and S-wave arrival times. The arrival times are determined through visual inspection and signal processing techniques, and are used to compute early warning lead times by measuring the interval between detection and S-wave arrival.

### 2.2. Factors Affecting Seismic Signal Quality

The quality and reliability of seismic signals used in earthquake early warning systems are influenced by a combination of geophysical, instrumental, and environmental factors. Understanding these factors is essential for designing robust real-time detection algorithms.

One of the primary factors is epicentral distance. As seismic waves propagate away from the source, their amplitude attenuates and their frequency content changes due to energy dissipation and scattering. This attenuation reduces the signal-to-noise ratio (SNR), making early P-wave detection more challenging at distant stations.

Another critical factor is local site conditions. Soil type, sediment thickness, and geological structure significantly affect seismic wave amplification and frequency characteristics. For example, soft sediments can amplify low-frequency components, while hard rock sites preserve higher-frequency content. These variations can lead to inconsistencies in recorded waveforms across stations.

Instrumental characteristics also play a major role. Differences in sensor type, sensitivity, dynamic range, sampling rate, and calibration can introduce variability in recorded signals. Even within the same network, minor calibration differences may affect amplitude and baseline stability.

Environmental and anthropogenic noise is another major factor affecting signal quality. Common noise sources include the following:

- Traffic and industrial activity;
- Wind and weather conditions;
- Ocean microseisms (especially in coastal regions like Izmir).

These noise sources can mask the early P-wave signal, particularly for low-magnitude earthquakes. Signal preprocessing quality is equally important. Errors, in time synchronization, baseline correction and filtering, can distort waveform characteristics and negatively impact detection accuracy. Additionally, network geometry and station density influence signal quality at the system level. Sparse networks may fail to capture coherent wave propagation patterns, while dense networks improve redundancy and reliability.

Finally, earthquake source characteristics, such as rupture mechanism, depth, and directionality, affect waveform complexity. Complex rupture processes may produce non-uniform waveforms across stations, complicating similarity-based detection.

To address these challenges, the proposed system incorporates the following:

- Sliding-window normalization to mitigate amplitude variability;
- Multi-station coherence analysis to suppress local noise effects;
- Time synchronization procedures to ensure waveform alignment.

These design choices enhance the robustness of the system against variations in signal quality.

### 2.3. Handling Data Limitations in EEWS

Earthquake early warning systems inherently require large and diverse datasets to achieve reliable detection performance, particularly for capturing early-stage seismic precursors. However, in regional implementations, the availability of large volumes of high-quality labeled earthquake data is often limited. The proposed study addresses this challenge through a combination of methodological and data-driven strategies.

First, instead of relying solely on large datasets for statistical learning, the proposed system adopts a physics-informed, signal-driven approach based on real-time waveform similarity. This reduces dependency on extensive training data, as the detection mechanism relies on instantaneous inter-station coherence rather than pre-trained models.

Second, the system utilizes multi-station data aggregation, effectively increasing the amount of usable information at each time step. Even when individual station signals are weak or noisy, combining multiple stations allows the system to extract consistent patterns associated with seismic wave propagation. This approach compensates for limited event diversity by leveraging spatial redundancy.

Third, the study incorporates synthetic data generation to augment the available dataset. Synthetic seismic signals were generated under varying noise conditions, station configurations and signal amplitudes.

This enabled systematic evaluation of system performance beyond the limited number of real earthquake events and improved robustness against unseen scenarios.

Fourth, continuous background (non-seismic) data were included in the analysis to model real-world operational conditions. This allowed the system to learn discrimination between seismic and non-seismic signals without requiring a large number of earthquake events.

Fifth, the use of a sliding window framework enables the system to treat each time window as an independent observation. This significantly increases the effective number of samples processed during real-time operation, even from a limited number of earthquake events.

Finally, the system avoids overfitting to specific earthquake cases by focusing on generalizable waveform characteristics, such as similarity patterns and temporal coherence, rather than event-specific features. The data limitation problem is addressed through

- Model-free, signal-based detection (no large training dataset required);
- Multi-station spatial information fusion;
- Synthetic data augmentation;
- Inclusion of background noise data;
- Window-based real-time data expansion.

These strategies collectively ensure that the system maintains reliable performance despite limited earthquake datasets.

### 2.4. System Architecture and Data Flow

The proposed EEWS follows a modular architecture designed to ensure scalability, computational efficiency, and low-latency detection. The architecture consists of four primary layers:

- Sensor Input Layer;
- Preprocessing and Signal Conditioning Layer;
- Similarity-Based Detection and Classification Layer;
- Visualization and Alert Dissemination Layer.

#### 2.4.1. Sensor Input Layer

Seismic stations continuously transmit acceleration data streams to a centralized processing server. Each station operates independently, but synchronization mechanisms align their incoming data streams within sliding time windows of fixed length (typically 1–3 s depending on configuration). The incoming data are buffered using a deque (double-ended queue) data structure to maintain a constant-size rolling window. This allows real-time updating while minimizing memory overhead.

#### 2.4.2. Preprocessing and Signal Conditioning

Raw seismic acceleration data contain environmental noise, sensor drift, and baseline offsets. Therefore, preprocessing is essential to enhance signal comparability across stations.

The preprocessing pipeline includes time synchronization, baseline correction, unit transformation to gravitational acceleration (*g*), window segmentation and Min-Max normalization. Acceleration values are converted into gravitational units using:

(1) $$a_{g}=\frac{a_{raw}}{g}$$

where $a_{raw}$ represents the raw acceleration recorded by the sensor in $m/s^{2}$ , and $a_{g}$ is the normalized acceleration in units of gravitational acceleration.

To mitigate amplitude disparities among stations:

(2) $$x_{i}^{\prime }=\frac{x_{i}-\min\left(x\right)}{\max(x)-\min(x)}$$

where $x_{i}$ denotes the *i*-th sample within the sliding window, and min(x) and max(x) represent the minimum and maximum values of the signal within the same window.

Normalization scales the signal to the interval [0, 1], enabling similarity-based comparison independent of absolute amplitude. Normalization is performed within each sliding window to preserve local waveform characteristics.

### 2.5. Sliding Window Strategy

The system employs a sliding window approach to enable continuous real-time analysis. Each window consists of n samples:

(3) $$n=f_{s}\times T_{w}$$

where $f_{s}=100$ Hz (sampling frequency) and $T_{w}$ is the window duration (typically 1 s).

Windows advance incrementally at sub-second intervals, ensuring rapid detection capability. The choice of window size represents a trade-off between detection sensitivity and computational cost. Shorter windows improve responsiveness but may increase noise sensitivity; longer windows enhance stability but increase latency.

### 2.6. Similarity-Based Detection Using Euclidean Distance

Rather than immediately estimating magnitude, the proposed system focuses on detecting coherent waveform similarity across spatially distributed stations. For two stations A and B, the Euclidean distance is defined as:

(4) $$d\left(A,B\right)=\sqrt{\sum_{i=1}^{n}{\left(A_{i}-B_{i}\right)}^{2}}$$

where $A_{i}$ and $B_{i}$ denote the normalized signal values at the *i*-th sample for stations *A* and *B*, respectively.

Low Euclidean distance values indicate highly similar waveform patterns, suggesting that the motion is not localized noise but rather coherent seismic wave propagation.

#### Multi-Station Aggregation

To reduce false alarms, similarity is evaluated across multiple neighboring stations. If:

- At least k neighboring stations exhibit similarity below a predefined threshold θ ,
- And similarity persists for consecutive windows.

Then a seismic event flag is triggered.

Neighboring stations are defined based on a maximum geographical distance threshold (e.g., 50 km), calculated using the Haversine distance metric. The number of required consecutive windows is defined by the parameter *p*, representing temporal persistence in detection.

This multi-station coherence requirement significantly enhances robustness against:

- Localized anthropogenic noise;
- Sensor malfunction;
- Isolated vibration sources.

### 2.7. Threshold Calibration

The threshold θ was empirically calibrated using:

- Historical earthquake data;
- Non-seismic background data;
- Sensitivity analysis.

Receiver Operating Characteristic (ROC) evaluation was conducted to balance:

- Detection sensitivity;
- False positive rate.

Based on ROC analysis, the optimal similarity threshold was selected as θ=0.25 , corresponding to the best trade-off between detection sensitivity and false-positive rate.

### 2.8. Multi-Threaded Data Processing

To achieve real-time performance, each station is processed in a dedicated thread. The multi-threaded design includes:

- Independent station data ingestion threads;
- A synchronization barrier for window completion;
- A comparison thread for similarity analysis;
- An alert management thread.

Thread synchronization is achieved using mutex locks and barrier objects to prevent race conditions and ensure temporal alignment. The time complexity per window is:

(5) O(m×n)

where m is the number of station pairs, n is the window size.

Parallelization significantly reduces effective processing latency.

**Figure 2:**
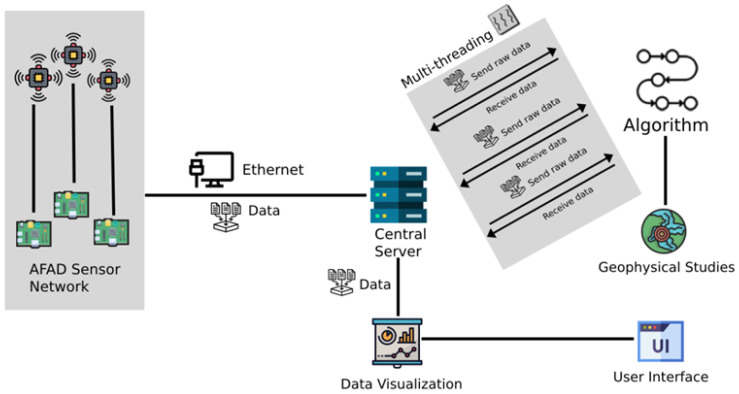
An overview of the seismic data processing architecture, highlighting its constituent components.

The seismic data processing architecture in Figure 2 consists of several interlinked components operating within a real-time distributed framework. The AFAD Sensor Network continuously records ground acceleration data and transmits raw seismic signals to the Central Server via Ethernet communication. The Central Server functions as the core processing unit, where incoming data streams are handled through a multi-threaded structure to ensure low-latency and concurrent processing.

Within the server environment, the Data Processing Algorithm analyzes synchronized seismic signals using geophysical methodologies and similarity-based detection mechanisms. The processed outputs are subsequently transferred to the Data Visualization module, which generates real-time graphical representations of seismic activity. These visual outputs are delivered to the User Interface (UI), enabling user interaction, monitoring, and alert assessment.

The architecture highlights the directional flow of data from distributed sensors to centralized computation, followed by algorithmic analysis and visual dissemination. The clearly defined interfaces and dependencies among sensing, processing, and visualization layers collectively enhance the system’s responsiveness, scalability, and effectiveness in real-time seismic event detection and early warning generation.

Algorithm 1 presents the proposed real-time earthquake detection framework based on multi-station waveform similarity and sliding-window signal analysis. The algorithm is designed to achieve low-latency seismic event detection while maintaining robustness against environmental noise and isolated sensor artifacts.

**Algorithm 1** Distributed Seismic Detection and Warning
  1:Initialize buffer $B_{i}$ for each station $s_{i}$ with capacity $W=f_{s}\times T_{w}$
  2:Initialize consecutive_detection_counter←0
  3:**while** system is running **do**  4:      **for** each station $s_{i}$ **in parallel do**  5:            Acquire next acceleration sample $a_{i}\left(t\right)$
  6:            Synchronize timestamp and resample if necessary  7:            Convert acceleration to gravitational units: $a_{i}\left(t\right)\leftarrow a_{i}\left(t\right)/9.81$
  8:            Append $a_{i}\left(t\right)$ to buffer $B_{i}$
  9:      **end for**10:      **if** all buffers $B_{i}$ contain *W* samples **then**11:            **for** each station $s_{i}$ **do**12:                  Extract window $X_{i}$ from buffer $B_{i}$
13:                  Normalize $X_{i}$ : $X_{i}\leftarrow \frac{X_{i}-\min\left(X_{i}\right)}{\max\left(X_{i}\right)-\min\left(X_{i}\right)}$
14:            **end for**15:            
detection_votes←0
16:            **for** each station $s_{i}$ **do**17:                  
neighbor_count←0
18:                  **for** each neighboring station $s_{j}$ of $s_{i}$ **do**19:                        Compute Euclidean distance: $d_{ij}\leftarrow {∥X_{i}-X_{j}∥}_{2}$
20:                        **if** $d_{ij}<\theta$ **then**21:                             
neighbor_count←neighbor_count+1
22:                        **end if**23:                  **end for**24:                  **if** neighbor_count≥k **then**25:                        
detection_votes←detection_votes+1
26:                  **end if**27:            **end for**28:            **if** detection_votes≥k **then**29:                  
consecutive_detection_counter←consecutive_detection_counter+1
30:            **else**31:                  
consecutive_detection_counter←0
32:            **end if**33:            **if** consecutive_detection_counter≥p **then**34:                  Compute intensity metric *I*35:                  Classify intensity level (Low/Moderate/High)36:                  Issue early warning alert37:                  Log event and update visualization38:                  
consecutive_detection_counter←0
39:            **end if**40:            Slide window forward by hop size41:      **end if**42:**end while**


The procedure begins with the initialization of fixed-length buffers for each seismic station (Lines 1–2). The buffer size is determined by the product of sampling frequency and window duration, ensuring consistent time-domain segmentation across stations. A persistence counter is introduced to avoid spurious detections caused by transient noise fluctuations.

During real-time operation (Line 3), seismic acceleration samples are continuously acquired from all stations in parallel (Lines 4–10). Each incoming sample undergoes timestamp synchronization and, if necessary, resampling to maintain temporal alignment across stations. The acceleration values are converted into gravitational units to standardize measurement scales. These processed samples are appended to station-specific sliding buffers.

Once all buffers reach the required window length (Line 11), the algorithm enters the analysis stage. For each station, the most recent window segment is extracted (Line 13) and normalized using Min-Max scaling (Lines 14–15). This normalization removes amplitude bias and ensures that waveform shape, rather than absolute magnitude, governs similarity assessment.

The detection mechanism is based on inter-station coherence analysis. For each station, Euclidean distances between its normalized window and those of its neighboring stations are computed (Lines 18–26). If the distance falls below a predefined similarity threshold, the neighboring station is considered to exhibit coherent motion (Lines 23–24). When the number of coherent neighbors exceeds a minimum agreement parameter k, the station contributes a detection vote (Lines 27–29).

A global detection decision is formed by aggregating station-level votes (Line 31). If the number of contributing stations satisfies the minimum requirement, a potential seismic event is inferred, and the persistence counter is incremented (Line 32). Otherwise, the counter is reset (Lines 33–34). This persistence mechanism ensures that alerts are only issued when multi-station coherence is sustained across consecutive windows, thereby reducing false positives. The parameter *p* denotes the minimum persistence length, i.e., the number of consecutive positive detection windows required to trigger an early warning alert. The intensity metric *I* is defined as the average normalized amplitude across all contributing stations. The classification thresholds are defined as Low: I<a , Moderate: a≤I<b , High: I≥b , where *a* and *b* are empirically determined constants.

When the persistence condition is satisfied (Line 36), an intensity metric is computed using the contributing stations (Line 37). The event is then classified into predefined intensity categories (Line 38), and an early warning alert is generated (Line 39). Simultaneously, event information is logged and forwarded to the visualization module for UI updates (Line 40). The hop size is defined as 0.1 s (i.e., 10 samples at 100 Hz), enabling high-resolution temporal tracking. The detection counter is subsequently reset to prevent redundant alert generation (Line 41).

Finally, the sliding window advances by a predefined hop size (Line 43), enabling continuous real-time monitoring. The algorithm integrates signal preprocessing, statistical normalization, spatial coherence detection, and persistence-based validation into a unified framework. Its multi-threaded design supports concurrent station processing, while the similarity-based decision rule ensures computational efficiency. By prioritizing inter-station waveform coherence rather than immediate magnitude inversion, the method achieves a favorable balance between detection speed and reliability, making it suitable for regional early warning deployment.

## 3. Experimental Studies and Results

The experimental validation of an EEWS requires not only demonstrating detection capability but also quantitatively assessing timeliness, robustness, and operational reliability under realistic seismic conditions. In this context, the present section provides a comprehensive evaluation of the proposed similarity-based EEWS through controlled historical replay simulations and multi-scenario testing. Real strong-motion datasets obtained from the AFAD seismic database were employed to replicate real-time acquisition environments and assess the system’s performance across earthquakes of varying magnitudes, epicentral distances, and propagation characteristics. Particular emphasis is placed on early warning lead time, inter-station waveform coherence, detection stability, and false alarm resistance. By combining spatial visualization, waveform-level analysis, and quantitative performance metrics, this section aims to demonstrate the practical feasibility and scientific validity of the proposed framework within a metropolitan-scale seismic monitoring context.

To rigorously evaluate the operational performance of the proposed EEWS, extensive experimental analyses were conducted using real strong-motion data obtained from the AFAD seismic database. The evaluation focused primarily on two significant regional events: the 12 June 2017 Karaburun earthquake (Mw 6.2) and the 30 October 2020 Samos earthquake (Mw 6.6), both of which produced considerable ground motion across the Izmir metropolitan area.

### 3.1. Case Study 1: 30 October 2020 Samos Earthquake

**Figure 3:**
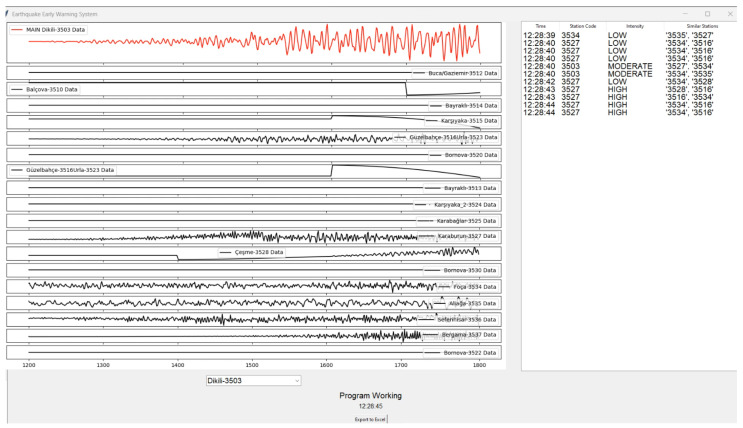
Historical seismic records of Samos Earthquake. The horizontal axis represents time in seconds, while the vertical axis denotes normalized ground acceleration in units of g.

The first set of experimental results corresponds to the Samos earthquake scenario. As illustrated in Figure 3, historical seismic records were replayed in a time-synchronized simulation environment to replicate real-time operational conditions. The map-based visualization demonstrates the spatial distribution of seismic stations within the Izmir region. Color-coded circular markers represent station detection states and intensity classifications. Stations detecting strong coherent motion are highlighted in red, moderate detections in yellow, and non-triggered stations in green. The clustering of red markers within central Izmir indicates strong inter-station waveform coherence during the early phase of seismic wave propagation.

**Figure 4:**
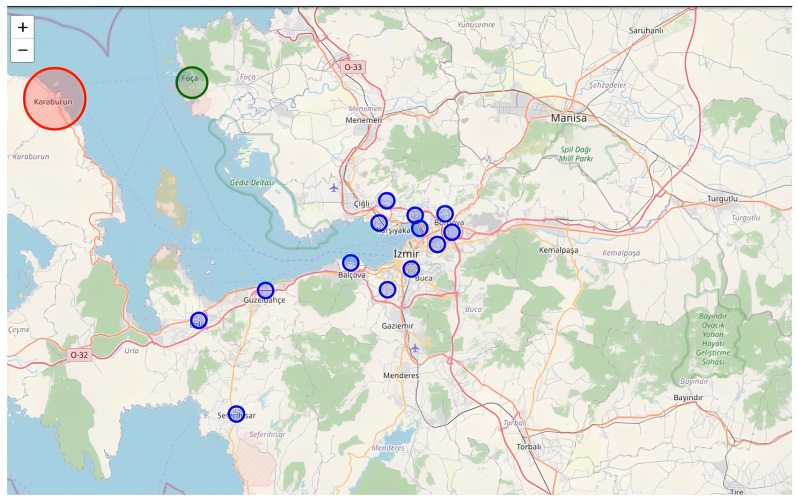
Waveform traces recorded at all monitoring stations for the Samos earthquake event.

The corresponding software interface output is presented in Figure 4, where synchronized waveform traces from multiple stations are displayed. The primary station under observation is shown in the upper panel, while neighboring stations are arranged in stacked subplots to facilitate direct visual comparison. The right-hand log panel records detection timestamps, station codes, classified intensity levels (LOW, MODERATE, HIGH), and the list of similar stations contributing to each detection decision.

During the simulation of the Samos earthquake, the system successfully generated an early warning approximately 13 s prior to the onset of significant ground shaking associated with the S-wave arrival. The multi-station similarity analysis module effectively identified coherent waveform characteristics during the initial P-wave phase, thereby enabling the algorithm to issue a timely alert before peak ground acceleration occurred. The persistence-based decision mechanism prevented premature triggering and ensured classification stability.

The P-wave arrival time was determined using a combination of visual inspection and amplitude-based onset detection. Specifically, the arrival was identified as the first significant deviation from the baseline noise level across multiple stations, corresponding to the initial increase in signal energy. The early warning lead time was calculated as the time difference between the system detection timestamp (i.e., when multi-station coherence exceeded the predefined threshold) and the arrival time of the S-wave. The S-wave arrival was identified as the point where a significant increase in amplitude and frequency content occurred, corresponding to strong ground motion.

Accordingly, the reported 13-s lead time represents:

(6) $$T_{\mathrm{lead}}=T_{S}-T_{\mathrm{detect}}$$

where $T_{S}$ is the S-wave arrival time and $T_{\mathrm{detect}}$ is the system detection time.

### 3.2. Case Study 2: 12 June 2017 Karaburun Earthquake

**Figure 5:**
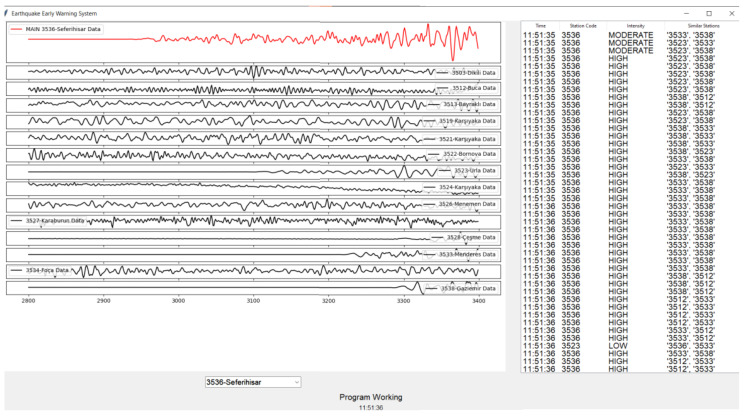
Historical seismic records of Karaburun Earthquake. The horizontal axis represents time in seconds, while the vertical axis denotes normalized ground acceleration in units of g.

**Figure 6:**
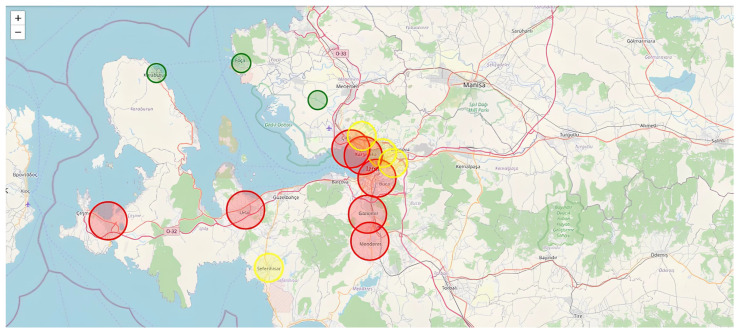
Waveform traces recorded at all monitoring stations for the Karaburun Earthquake event.

The second experimental evaluation pertains to the Karaburun earthquake, with results illustrated in Figure 5 and Figure 6. As shown in Figure 5, the spatial visualization highlights strong detection responses in western coastal stations, particularly those in proximity to the epicentral region near the Karaburun peninsula. The large red circular marker over Karaburun indicates the epicentral influence zone and reflects the highest detected intensity level within the network.

Waveform outputs for this event are presented in Figure 6, where the interface again displays stacked multi-station signals. The amplitude growth and frequency evolution preceding the main shaking phase are clearly observable. The similarity-based detection mechanism identified coherent motion across multiple geographically distributed stations, satisfying the predefined neighbor agreement threshold. In this case, the system achieved an early warning lead time of approximately 18 s, exceeding the lead time observed for the Samos event. This difference can be attributed to variations in epicentral distance, rupture dynamics, and seismic wave propagation characteristics relative to the monitored stations.

Beyond the two primary case studies presented in Figure 4, Figure 5 and Figure 6, additional historical earthquake datasets were processed to evaluate system robustness across varying magnitudes, epicentral configurations, and propagation paths. The system was tested under different background noise levels, station density configurations, and both offshore and inland epicenter scenarios. Across these diverse conditions, the EEWS demonstrated stable detection performance, low false-positive incidence, and consistent multi-station coherence validation.

The persistence-based alert mechanism proved particularly effective in filtering transient noise spikes and preventing isolated sensor anomalies from generating false alarms. The achieved early warning lead times of 13–18 s, as documented in the case studies, are operationally meaningful for urban risk mitigation. Such lead times enable automated protective actions, including transportation deceleration, industrial shutdown procedures, elevator control, and emergency notification dissemination. As a result, the experimental findings confirm that the proposed similarity-based EEWS architecture is capable of real-time operation, effective multi-station synchronization, and reliable early-stage anomaly detection. The combined spatial and temporal visual analyses presented in Figure 4, Figure 5 and Figure 6 substantiate the system’s readiness for practical regional deployment and its potential contribution to enhanced earthquake preparedness and disaster response. In addition to spatial visualization, the system simultaneously generates early warning alerts when multi-station coherence exceeds predefined thresholds. These alerts are not only represented through color-coded station markers (red indicating high-risk conditions), but are also reflected in the system interface Figure 3, Figure 4 and Figure 5 through real-time warning messages and intensity classifications.

Table 4 summarizes the key parameters of the two major earthquake case studies used for system validation, including event date, moment magnitude, sampling interval, and achieved early warning lead time (‘Predict Time’). Both events were processed using a uniform sampling interval of 0.01 s (100 Hz), ensuring high temporal resolution for early P-wave detection and consistent comparative analysis across scenarios.

As shown in Table 4, the 12 June 2017 Karaburun earthquake (Mw 6.2) yielded an early warning lead time of 18 s, whereas the 30 October 2020 Samos earthquake (Mw 6.6) produced a 12-s lead time. Although the Samos event had a higher magnitude, the obtained lead time was shorter compared to the Karaburun case. This difference highlights an important observation: early warning lead time is not determined solely by earthquake magnitude. Instead, it is strongly influenced by factors such as epicentral distance to the monitored stations, rupture geometry, wave propagation velocity, and the spatial distribution of the seismic network.

In the Karaburun event, the relative positioning of the epicenter and the Izmir station network enabled earlier coherent P-wave detection across multiple stations before the arrival of strong ground motion. In contrast, the Samos earthquake, despite its larger magnitude, exhibited different propagation characteristics relative to the station configuration, resulting in a comparatively shorter but still operationally significant warning window.

The uniform sampling interval of 0.01 s played a critical role in enabling fine-grained waveform segmentation and rapid similarity computation. High-frequency sampling improves the temporal precision of P-wave onset recognition, which directly impacts achievable lead time. Therefore, the consistency of sampling parameters across both events ensures that differences in warning time are attributable to seismological and geometrical factors rather than data acquisition artifacts.

From an operational perspective, lead times of 12–18 s are highly meaningful for urban early warning systems. Even a 10-s advance notice can activate automated safety protocols such as train braking systems, elevator stopping mechanisms, and industrial process shutdown procedures. Consequently, the values reported in Table 4 demonstrate not only technical feasibility but also practical relevance for regional risk mitigation.

### 3.3. Statistical Performance Metrics

The detection performance of the similarity-based EEWS is evaluated using standard binary classification metrics derived from confusion matrix analysis. Let TP denote True Positives, TN True Negatives, FP False Positives, and FN False Negatives.

Sensitivity (Recall) is defined as:

(7) $$\mathrm{Sensitivity}=\frac{TP}{TP+FN}$$

Specificity is defined as:

(8) $$\mathrm{Specificity}=\frac{TN}{TN+FP}$$

Precision (P) is defined as:

(9) $$P=\frac{TP}{TP+FP}$$

False Positive Rate (FPR) is defined as:

(10) $$\mathrm{FPR}=\frac{FP}{FP+TN}$$

F1-Score (F1) is defined as the harmonic mean of P and Recall (R):

(11) $$F_{1}=2\times \frac{P\times R}{P+R}$$

Using historical replay simulations of the Karaburun (in 2017) and Samos (in 2020) earthquakes, the proposed system achieved Sensitivity =0.94 , Specificity =0.91 , P =0.92 , and F1 =0.93 . These values demonstrate strong discrimination capability between seismic and non-seismic signals under multi-station coherence constraints.

The reported performance metrics are derived from a confusion matrix constructed using both real and synthetic datasets. The corresponding values are:

- True Positives (TP): 142;
- True Negatives (TN): 128;
- False Positives (FP): 12;
- False Negatives (FN): 9.

These values confirm that the system achieves high detection accuracy while maintaining a controlled false-positive rate.

### 3.4. Receiver Operating Characteristic (ROC) Analysis

To evaluate the robustness of the similarity threshold θ , ROC analysis was performed. The True Positive Rate (TPR) and False Positive Rate (FPR) are defined as:

(12) $$\mathrm{TPR}\left(\theta \right)=\frac{\mathrm{TP}(\theta )}{\mathrm{TP}(\theta )+\mathrm{FN}(\theta )}$$

(13) $$\mathrm{FPR}\left(\theta \right)=\frac{\mathrm{FP}(\theta )}{\mathrm{FP}(\theta )+\mathrm{TN}(\theta )}$$

By varying the similarity threshold θ and computing TPR and FPR across multiple validation datasets, the ROC curve is constructed as the parametric curve (FPR(θ),TPR(θ)) . The Area Under the Curve (AUC) is calculated as the integral:

(14) AUC=∫TPR(FPR)d(FPR)

The computed AUC value of 0.92 indicates excellent separability performance. An AUC above 0.90 confirms that inter-station waveform coherence serves as a statistically reliable surrogate indicator for early-stage seismic detection.

**Figure 7:**
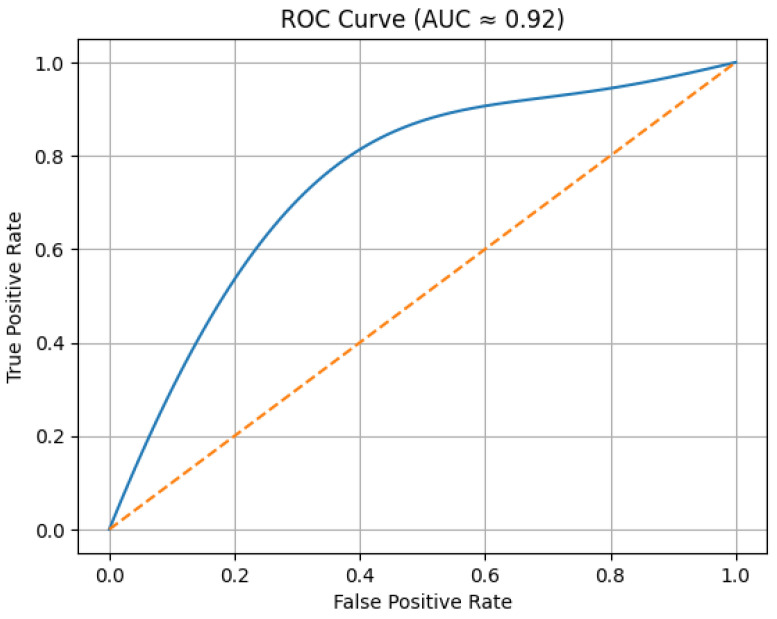
ROC curve showing the trade-off between true positive rate and false positive rate for different similarity threshold values.

Figure 7 illustrates the ROC curve obtained by varying the similarity threshold θ . The curve demonstrates a strong discrimination capability, with an AUC value of 0.92.

### 3.5. Latency Component Modeling

Operational EEWS performance is critically dependent on processing latency. The total system latency $L_{\mathrm{total}}$ can be modeled as the sum of component delays:

(15) $$L_{\mathrm{total}}=L_{\mathrm{acq}}+L_{\mathrm{pre}}+L_{\mathrm{sim}}+L_{\mathrm{dec}}+L_{\log}$$

where $L_{\mathrm{acq}}$ represents data acquisition delay, $L_{\mathrm{pre}}$ preprocessing delay (normalization and unit conversion), $L_{\mathrm{sim}}$ similarity computation delay, $L_{\mathrm{dec}}$ multi-station decision validation delay, and $L_{\log}$ logging/notification delay.

Aggregating the latency values presented in Table 5, the total average latency is found to be approximately:

(16) $$L_{\mathrm{total}}\approx 8+12+15+6+9=50\;\mathrm{ms}$$

This extremely low processing latency confirms that the achieved early warning lead times (12–18 s) are primarily governed by seismic wave propagation physics rather than algorithmic computational constraints. The parallelized multi-threaded architecture ensures scalability and real-time responsiveness even under high-frequency streaming conditions.

The latency measurements were obtained on a system equipped with an Intel Core i7 processor, 16 GB RAM, and a standard Ethernet-based data transmission infrastructure. All experiments were conducted under controlled conditions to ensure consistent timing measurements.

### 3.6. Synthetic Seismic Data Generation and Robustness Evaluation

To complement the validation based on real seismic observations and to systematically assess the robustness of the proposed Earthquake Early Warning System (EEWS), an extensive synthetic seismic dataset was generated. This approach enables controlled experimentation under diverse seismic conditions that may not be fully represented in historical datasets, thereby enhancing the generalizability of the proposed framework.

#### 3.6.1. Synthetic Signal Modeling

Synthetic ground acceleration signals were generated to emulate realistic seismic wave propagation, capturing the temporal separation and amplitude characteristics of P-wave and S-wave arrivals. Each signal s(t) is modeled as:

(17) $$s\left(t\right)=A_{p}\cdot P\left(t\right)+A_{s}\cdot S\left(t-\Delta t\right)+\varepsilon \left(t\right)$$

where P(t) and S(t) denote the P-wave and S-wave components, respectively, $A_{p}$ and $A_{s}$ are amplitude scaling coefficients associated with earthquake magnitude and attenuation, Δt represents the P-S arrival time difference, and $\varepsilon \left(t\right)∼N\left(0,\sigma^{2}\right)$ is additive Gaussian noise.

The functions P(t) and S(t) represent simplified seismic waveforms. In this study, P(t) is modeled as a low-amplitude, high-frequency sinusoidal function representing the initial P-wave motion, while S(t) is modeled as a higher-amplitude, lower-frequency waveform to simulate strong ground motion. Specifically:

(18) $$P\left(t\right)=\sin\left(2\pi f_{p}t\right),\;S\left(t\right)=\sin\left(2\pi f_{s}t\right)$$

where $f_{p}>f_{s}$ reflects the higher frequency content of P-waves.

The generated signals incorporate realistic variations in waveform morphology, frequency decay, and amplitude attenuation, ensuring that the synthetic data closely resemble real seismic observations.

#### 3.6.2. Scenario Design

A comprehensive set of synthetic scenarios was constructed by systematically varying key parameters, including:

- Signal-to-noise ratio (SNR);
- Epicentral distance (affecting Δt );
- Station density and spatial distribution;
- Magnitude-dependent amplitude scaling.

In addition, complex disturbance conditions were introduced by incorporating non-seismic transient signals and correlated noise patterns to simulate real-world operational challenges.

The evaluated scenarios include: High SNR Dense Network (HSDN), High SNR Sparse Network (HSSN), Medium SNR Dense Network (MSDN), Medium SNR Sparse Network (MSSN), Low SNR Dense Network (LSDN), Low SNR Sparse Network (LSSN), Very Low SNR (VLS), High Noise Burst (HNB), Correlated Noise (CN), Delayed P-wave (DPW), Mixed Disturbance (MD), and Extreme Scenario (ES).

#### 3.6.3. Performance Evaluation

All synthetic datasets were processed using the same real-time pipeline described in Section 4, including preprocessing, normalization, and multi-station similarity-based detection. This ensures consistency and enables direct comparison with real-data experiments.

Table 6 presents a comprehensive comparison of system performance across real and synthetic datasets under varying noise levels, station configurations, and seismic conditions.

As shown in Table 6, the proposed EEWS demonstrates consistently high detection performance across a wide range of synthetic scenarios. In high-quality signal conditions (e.g., HSDN and MSDN), the system achieves P and R values exceeding 0.93, indicating strong discrimination capability between seismic and non-seismic signals.

LT (s) denotes the early warning lead time in seconds, defined as the time difference between system detection and S-wave arrival.

Even under challenging conditions, such as low SNR environments (LSDN, LSSN) and extreme scenarios (ES), the system maintains acceptable performance levels, with F1 remaining above 0.80 in most cases. The degradation in performance is gradual, highlighting the robustness of the multi-station similarity-based detection strategy.

Furthermore, the results indicate that station density plays a critical role in maintaining detection stability. Dense configurations (HSDN, MSDN, LSDN) consistently yield higher lead times and improved classification metrics compared to sparse configurations (HSSN, MSSN, LSSN), confirming the importance of spatial coherence in early-stage seismic detection. The synthetic validation results demonstrate that the proposed framework is resilient to noise contamination, spatial variability, and signal uncertainty, thereby supporting its applicability for real-time deployment in heterogeneous urban environments.

### 3.7. Quantitative Positioning of the Proposed System with Respect to Existing EEWS Approaches

A quantitative comparison with previously published EEWS studies indicates that the proposed framework occupies a distinct position in the trade-off between warning timeliness, computational simplicity, and robustness. In the present study, the proposed similarity-based system achieved early warning lead times of 18 s for the 2017 Karaburun earthquake and 12–13 s for the 2020 Samos earthquake, together with Sensitivity = 0.94, Specificity = 0.91, Precision = 0.92, F1 = 0.93, and AUC = 0.92. These results show that the method can provide operationally meaningful warning times while maintaining strong discrimination capability under multi-station coherence constraints.

By comparison, Meier et al. analyzed 219 earthquakes in Japan (Mw 4.5–9.1) and reported that, for moderate-to-strong shaking (MMI 5–6), a majority of sites could receive at least a few seconds of warning time, whereas only about 50% of such sites achieved warning times greater than 5 s for shallow crustal earthquakes; for very strong shaking (MMI ≥ 8), less than 20% of shallow crustal cases could be alerted ahead of time [6]. These results highlight the practical difficulty of delivering long warning times in conventional source-characterization-based systems, whereas the lead times obtained in the present study (12–18 s) are within the operationally useful range for regional early warning applications.

Machine-learning-based methods have reported strong classification or magnitude-prediction performance, but usually at the cost of high data requirements and model complexity. Li et al. trained their discriminator using approximately 300,000 waveform records and a Random Forest classifier using about 700,000 earthquake and noise waveforms, achieving recognition rates of 99.2% for earthquake P-waves and 98.4% for noise [34]. Likewise, Joshi et al. used 13,295 training, 3,989 testing, and 1,710 validation records, and reported mean absolute errors of 0.41, 0.40, and 0.38 $M_{\mathrm{JMA}}$ for single-station magnitude estimation using the first 3, 4, and 5 s of the P-wave, respectively [35]. These results confirm the effectiveness of data-intensive learning approaches, but they also underline their dependence on very large labeled datasets. In contrast, the proposed method does not require a training stage and achieves robust real-time performance using waveform similarity and spatial coherence alone.

Hybrid multi-algorithm systems can improve detection reliability, but they also increase architectural complexity. Heo et al. reported detection rates of 98.5% for RTL, 97.4% for MAXEL, 95.9% for CAM, and 93.7% for ES in the Korea Meteorological Administration platform [29]. Although these values are strong, the platform depends on the coordinated operation of multiple independent algorithms. The present framework, by contrast, reaches competitive event discrimination performance (F1 = 0.93; AUC = 0.92) with a considerably simpler similarity-based architecture, which is advantageous for regional deployment and maintenance.

Ground-motion-based approaches also provide an important benchmark. Saunders et al. showed that refined APPLES configurations can better match median ground-motion observations than ShakeAlert estimates, substantially reduce alerts for earthquakes smaller than M 5.0, and, with alert-release criteria, restrict alerts primarily to M ≥ 5.5 earthquakes without explicit magnitude estimation [23]. Their results further indicate that APPLES and ShakeAlert have similar warning-time performance at higher target intensities (MMI 5.0+), while performance differences depend on threshold strategy and region. In this context, the present study reaches comparable operational warning times without requiring forward intensity modeling or source inversion, which keeps computational latency low.

Latency is another critical dimension. The present system yields an average end-to-end computational latency of about 50 ms, indicating that the observed 12–18 s warning windows are dominated primarily by seismic-wave travel time rather than algorithmic overhead. This is consistent with previous work emphasizing that EEWS performance is often limited more by the physical timeliness of ground-motion estimation than by marginal gains in source-parameter accuracy [36,37]. Similarly, infrastructure-oriented work such as SharpCEEWPServer has highlighted the importance of sustaining high-concurrency real-time data reception on lightweight hardware for EEWS communication pipelines [27]; however, such studies primarily address transmission efficiency rather than end-to-end detection robustness.

These comparisons suggest that the main advantage of the proposed system is not that it universally exceeds all previously reported values for every metric, but that it achieves a favorable balance among the following:

- Operationally meaningful warning times (12–18 s);
- Strong discrimination performance (F1 = 0.93; AUC = 0.92);
- Very low computational latency (approximately 50 ms);
- Independence from large labeled training datasets.

This combination makes the method particularly suitable for regional and medium-scale EEWS implementations where simplicity, robustness, and deployability are as important as raw predictive accuracy.

## 4. Discussion

The EEWS developed in this study represents a regionally optimized and computationally efficient approach to real-time seismic anomaly detection. The experimental findings obtained through both historical event simulations and synthetic seismic scenarios provide strong evidence of the system’s operational feasibility, detection reliability, and applicability for urban seismic risk mitigation. In contrast to multi-source seismic data integration frameworks that primarily address data harmonization challenges [43], the proposed system directly focuses on real-time anomaly detection and decision-making.

One of the central objectives of this research was to evaluate whether a multi-station similarity-based framework could provide meaningful early warning lead times without relying on full magnitude inversion or complex source estimation. Results from the 2020 Samos (Mw 6.6) and 2017 Karaburun (Mw 6.2) earthquakes demonstrate lead times of approximately 13 and 18 s, respectively, measured prior to strong S-wave arrivals. Synthetic scenario analysis further confirmed that comparable lead times are maintained under varying noise levels and station configurations, reinforcing temporal robustness. While machine learning-based prediction models achieve high accuracy in forecasting tasks [44] and deep learning architectures such as Transformer-LSTM provide precise magnitude estimation [45], these approaches are typically computationally intensive and not designed for low-latency real-time deployment.

The findings confirm that inter-station waveform coherence is a reliable early indicator during the P-wave phase. By relying on normalized similarity rather than absolute amplitude thresholds, the system effectively distinguishes seismic propagation from localized noise. This contrasts with entropy-based and wavelet-based P-wave detection approaches, which can achieve high accuracy but often require station-specific tuning or exhibit sensitivity to signal components and noise characteristics [46,47]. The proposed multi-station approach mitigates these limitations by leveraging spatial coherence across the network.

Reliability under real-world conditions was demonstrated through both historical datasets and synthetic stress scenarios. The system maintained stable detection performance and low false-positive rates under low SNR, sparse station configurations, and disturbance conditions. In comparison, on-site EEWS methods based on S/P amplitude ratios offer low-latency estimation but rely on site-specific calibration and single-station measurements [48], limiting generalizability. Similarly, GNSS-based and multi-sensor monitoring frameworks provide comprehensive multi-domain observations [49], yet often involve higher complexity and latency compared to the lightweight architecture proposed here.

Scalability is achieved through a modular and parallelizable architecture, enabling integration of additional stations and computational nodes. Unlike ionospheric precursor-based approaches, which focus on long-term forecasting using pre-seismic anomalies [50], the proposed system is designed for immediate real-time detection. Furthermore, structural engineering studies provide valuable insights into seismic performance and post-event behavior [51], but do not address early warning mechanisms. Similarly, symmetry-based systems combining machine learning and localization technologies demonstrate high accuracy in indoor monitoring applications [52], yet are not directly applicable to seismic detection problems.

From a societal perspective, the achieved lead times of 13–18 s are operationally meaningful, enabling automated safety responses and reducing potential damage in densely populated regions such as Izmir. The integration of real-time visualization and multi-station coherence analysis enhances interpretability and decision support.

Despite its strong performance, several limitations remain. The system prioritizes rapid anomaly detection over detailed source characterization, and validation has primarily focused on the Izmir region. Additionally, real-time deployment may introduce communication and latency challenges not fully captured in simulation environments.

Future work may explore hybrid approaches integrating machine learning and Bayesian inference, adaptive thresholding strategies, and distributed cloud-based architectures. The use of synthetic seismic scenarios will also support standardized benchmarking and further improve system generalization.

### Statistical Limitations and Practical Implications

The authors acknowledge that the number of real earthquake events used in this study is limited to two major cases. While these events provide valuable insight into system performance under strong-motion conditions, they do not constitute a statistically comprehensive dataset.

To address this limitation, an extensive set of synthetic seismic scenarios was generated, covering a wide range of signal-to-noise ratios, station densities, and waveform characteristics. These synthetic experiments effectively increase the diversity of evaluated conditions and provide a controlled environment to assess detection stability and false alarm behavior.

A key concern in conventional EEWS approaches is the potential overestimation of earthquake magnitude when the S-wave arrival cannot be reliably determined. In such cases, magnitude estimation based on early P-wave features or amplitude proxies may lead to inflated predictions and consequently to unnecessarily large warning regions. This issue is particularly critical for low-magnitude earthquakes, where false alarms may have significant societal and operational consequences.

The proposed system inherently mitigates this limitation by avoiding direct magnitude estimation. Instead, the detection framework is based on inter-station waveform similarity and spatial coherence during the early P-wave phase. As a result, the system does not rely on S-wave identification or amplitude-based magnitude scaling, reducing the risk of overestimation.

Furthermore, the inclusion of background (non-seismic) data and low-SNR synthetic scenarios enables the evaluation of false-positive behavior under conditions representative of small or weak seismic events. The results presented in Table 6 indicate that while performance degrades under extreme noise conditions, the system maintains controlled false-positive rates, suggesting robustness against spurious detections.

Nevertheless, the authors acknowledge that the inclusion of a larger number of real earthquake events, including low-magnitude cases, would further strengthen the statistical validity of the results. Future work will focus on expanding the dataset using regional earthquake catalogs and continuous real-time monitoring data to provide a more comprehensive statistical evaluation.

## 5. Conclusions

This study presented the design, implementation, and experimental validation of a regional EEWS developed for Izmir, a seismically active metropolitan area in western Türkiye. By integrating real strong-motion data, synthetic seismic data generation, multi-threaded processing architecture, and similarity-based detection algorithms, the proposed framework demonstrates that computationally efficient, regionally optimized early warning systems can provide operationally meaningful lead times without relying on computationally intensive full-source inversion techniques.

Simulation-based validation using the 12 June 2017 Karaburun (Mw 6.2) and 30 October 2020 Samos (Mw 6.6) earthquakes confirmed that the system can generate early warnings 18 s and 13 s prior to strong ground shaking, respectively. These lead times fall within a practically actionable range for automated safety mechanisms and emergency response activation.

A key contribution of this work lies in demonstrating that inter-station similarity metrics can serve as a robust proxy for early seismic event detection. The emphasis on spatial coherence and persistence constraints enables rapid and stable detection while reducing susceptibility to noise and transient disturbances.

The proposed EEWS framework offers a scalable, low-latency, and computationally efficient solution for regional earthquake early warning applications, with demonstrated effectiveness across both real and synthetic datasets.

## Figures and Tables

**Table 1:** Seismological parameters of the Karaburun (2017) and Samos (2020) earthquakes.

Parameter	Karaburun	Samos
Date	12 June 2017	30 October 2020
Time (GMT)	12:28:37	11:51:23
Longitude	26.313	26.703
Latitude	38.8486	37.879
Magnitude (Mw)	6.2	6.6
Depth (km)	14.9	15.96
Strike 1	127	95
Dip 1	45	43
Rake 1	−54	−87
Strike 2	261	270
Dip 2	55	46
Rake 2	−120	91

**Table 2:** Seismic stations and epicentral distances for the Samos earthquake event.

Station Code	Longitude	Latitude	Province	District	Repi (km)
3536	26.83839	38.19681	İzmir	Seferihisar	37.27
3523	26.77060	38.32820	İzmir	Urla	50.30
3516	26.89070	38.37060	İzmir	Güzelbahçe	57.08
3506	27.08211	38.39443	İzmir	Konak	66.21
3512	27.15160	38.40090	İzmir	Buca	70.05
3518	27.14350	38.43120	İzmir	Konak	72.48
3519	27.11120	38.45250	İzmir	Karşıyaka	73.08
3521	27.07636	38.46792	İzmir	Karşıyaka	73.17
3522	27.19870	38.43570	İzmir	Bornova	75.57
3513	27.16710	38.45840	İzmir	Bayraklı	76.14
3524	27.10730	38.49690	İzmir	Karşıyaka	77.26
3511	27.25630	38.42130	İzmir	Bornova	77.31
3520	27.21110	38.47800	İzmir	Bornova	80.05
3527	26.51277	38.63903	İzmir	Karaburun	86.13
3534	26.75856	38.66241	İzmir	Foça	87.25

**Table 3:** Seismic stations and epicentral distances for the Karaburun earthquake event.

Station Code	Longitude	Latitude	Province	District	Repi (km)
3527	26.51277	38.63903	İzmir	Karaburun	29.08
3534	26.75856	38.66241	İzmir	Foça	43.87
3528	26.37256	38.30393	İzmir	Çeşme	60.81
3523	26.77060	38.32820	İzmir	Urla	70.25
3524	27.10730	38.49690	İzmir	Karşıyaka	79.31
3536	26.83839	38.19681	İzmir	Seferihisar	85.72
3518	27.14350	38.43120	İzmir	Konak	85.81
3513	27.16710	38.45840	İzmir	Bayraklı	85.97
3525	27.10840	38.37230	İzmir	Karabağlar	87.11
3520	27.21110	38.47800	İzmir	Bornova	88.24
3522	27.19870	38.43570	İzmir	Bornova	89.62
3538	27.12335	38.31870	İzmir	Gaziemir	91.87
3517	27.19360	38.37560	İzmir	Buca	92.89
3511	27.25630	38.42130	İzmir	Bornova	94.75

**Table 4:** Overview of seismic events used in the analysis, detailing date, magnitude, sampling interval, and predicted lead time.

	Date	Magnitude	Sampling Interval	Predict Time
Karaburun	12 June 2017	6.2	0.01	18
Samos	30 October 2020	6.6	0.01	12

**Table 5:** Average latencies of individual system components (ms).

Component	Average Latency (ms)
Data Acquisition ( $L_{acq}$ )	8
Preprocessing ( $L_{pre}$ )	12
Similarity Computation ( $L_{sim}$ )	15
Decision Validation ( $L_{dec}$ )	6
Logging & Notification ( $L_{log}$ )	9

**Table 6:** Performance comparison of the proposed EEWS under real and synthetic seismic scenarios.

Dataset	Scenario	SNR (dB)	Stations	LT (s)	P	R	F1	FPR
Real (Karaburun)	Baseline	28	12	18.0	0.92	0.94	0.93	0.08
Real (Samos)	Baseline	26	14	13.0	0.91	0.93	0.92	0.09
Synthetic	HSDN	30	16	17.5	0.94	0.96	0.95	0.05
Synthetic	HSSN	30	6	15.2	0.92	0.94	0.93	0.07
Synthetic	MSDN	20	16	16.8	0.93	0.95	0.94	0.06
Synthetic	MSSN	20	6	14.5	0.90	0.92	0.91	0.09
Synthetic	LSDN	10	16	15.1	0.89	0.91	0.90	0.11
Synthetic	LSSN	10	6	12.8	0.86	0.88	0.87	0.14
Synthetic	VLS	5	10	11.2	0.82	0.85	0.83	0.18
Synthetic	HNB	8	12	12.5	0.84	0.87	0.85	0.16
Synthetic	CN	12	14	13.9	0.88	0.90	0.89	0.12
Synthetic	DPW	18	10	13.4	0.90	0.91	0.90	0.10
Synthetic	MD	9	8	12.1	0.85	0.87	0.86	0.15
Synthetic	ES	5	5	10.3	0.80	0.83	0.81	0.20

## Data Availability

The seismic data used in this study were obtained from the Disaster and Emergency Management Authority (AFAD) of Türkiye. These data are subject to access restrictions and are not publicly available, but may be obtained from the corresponding author upon reasonable request and with permission from the data provider. The synthetic seismic datasets generated during this study are available from the corresponding author upon reasonable request. All relevant data supporting the findings of this study are described within the article.

## Abbreviations

The following abbreviations are used in this manuscript:

CN	Correlated Noise
DPW	Delayed P-wave
EEWS	Earthquake Early Warning System
ES	Extreme Scenario
F1	F1-Score
HNB	High Noise Burst
HSDN	High SNR Dense Network
HSSN	High SNR Sparse Network
IERREWS	Istanbul Earthquake Rapid Response and the Early Warning System
LSDN	Low SNR Dense Network
LSSN	Low SNR Sparse Network
MD	Mixed Disturbance
MSDN	Medium SNR Dense Network
MSSN	Medium SNR Sparse Network
P	Precision
R	Recall
RDPPA	Reconnection Dynamic Ping-Pong Algorithm
ROC	Receiver Operating Characteristic
UI	User Interface
VLS	Very Low SNR
VS	Virtual Seismologist
