# Correction: Doğan et al. Earthquake Early Warning System for Izmir, Western Anatolia, Türkiye Based on Multi-Station Similarity Analysis and Real-Time Seismic Data Processing. *Sensors* **2026**, *26*, 2931

**DOI:** 10.3390/s26113337

**Published:** 2026-05-25

**Authors:** Yunus Doğan, Ahmet Başbuğ, Fatih Semirgin, Yusuf Eren Kaya, Orkun Çınar, Hasan Sözbilir, Özkan Cevdet Özdağ, Reyat Yılmaz, Alp Kut, Özgür Tamer, Recep Çakır, Mehmet Utku, Özgür Özçelik, Mustafa Softa

**Affiliations:** 1 Department of Computer Engineering, Dokuz Eylül University, Izmir 35390, Türkiye; ahmet.basbug@ogr.deu.edu.tr (A.B.); fatih.semirgin@ogr.deu.edu.tr (F.S.); yusuf.kaya@ogr.deu.edu.tr (Y.E.K.); orkun@cs.deu.edu.tr (O.Ç.); alp@cs.deu.edu.tr (A.K.); 2 Department of Geological Engineering, Dokuz Eylül University, Izmir 35390, Türkiye; hasan.sozbilir@deu.edu.tr (H.S.); mustafa.softa@deu.edu.tr (M.S.); 3 Earthquake Research and Application Center, Dokuz Eylül University, Izmir 35390, Türkiye; 4 Department of Geophysical Engineering, Dokuz Eylül University, Izmir 35390, Türkiye; cevdet.ozdag@deu.edu.tr (Ö.C.Ö.); mehmet.utku@deu.edu.tr (M.U.); 5 Department of Electrical and Electronics Engineering, Dokuz Eylül University, Izmir 35390, Türkiye; reyad.yilmaz@deu.edu.tr (R.Y.); ozgur.tamer@deu.edu.tr (Ö.T.); 6 Civil Engineering Department, İzmir Institute of Technology, Izmir 35430, Türkiye; recep.cakir@dnr.wa.gov; 7 Civil Engineering Department, Dokuz Eylül University, Izmir 35390, Türkiye; ozgur.ozcelik@deu.edu.tr

**Affiliation Correction**

In the original publication [1], the content in Affiliation 6, “Civil Engineering Department, Izmir Institute of Technology, Izmir 35390, Türkiye; recep.cakir@dnr.wa.gov” has been updated to “Civil Engineering Department, İzmir Institute of Technology, Izmir 35430, Türkiye; recep.cakir@dnr.wa.gov”.

**Figure/Table Legend**

In Figure 1, the caption content “(**a**) Tectonic setting of the Western Anatolia Extensional Province (WAEP); (**b**) surrounding major fault systems.” has been updated to “(**a**) Tectonic setting of the Western Anatolia Extensional Province (WAEP). The İzmir-Balıkesir Transfer Zone (IBTZ) defines the boundary between the normal-fault-dominated West Anatolian Extensional Province (WAEP) and the strike-slip-dominated North Aegean Region (NAR). Abbreviations: NAFZ, North Anatolian Fault Zone; AA, Aegean Arc; CA, Cyprian Arc. Modified from Bozkurt (2001) [40] and Özkaymak et al. (2013) [41]. (**b**) Surrounding major fault systems. Compiled from Emre et al. (2018) [42].”

**Missing Funding**

In Funding, the content “This study was supported under the Dokuz Eylul University Scientific Research Projects program, under grant number 2020.KB.MLT.009 and titled “Development of an Earthquake Early Warning System (DEUSIS) for Izmir: Sensor, Methodology, and Forecasting Studies” has been updated to “This study was supported by the Dokuz Eylül University Scientific Research Projects program, under grant numbers 2020.KB.MLT.009, FBA-2022-2929 and FBA-2024-3400 and titled “Development of an Earthquake Early Warning System (DEUSIS) for Izmir: Sensor, Methodology, and Forecasting Studies”, “Determining standards for Earthquake Master Plans for Türkiye (TÜRDEMAP): İzmir city as an example”—Phase-1 and 2, respectively.”

**Acknowledgements Correction**

In Acknowledgements, the content “This study is derived from the undergraduate theses of Ahmet Başbuğ, Fatih Semirgin, and Yusuf Eren Kaya. The authors would like to express their sincere appreciation for their contributions to this work. The authors also gratefully acknowledge the valuable support and guidance provided by Orkun Çınar during the development of this study.” has been updated to “This study is derived from projects 2020.KB.MLT.009, FBA-2022-2929, and FBA-2024-3400 and the undergraduate theses of Ahmet Başbuğ, Fatih Semirgin, and Yusuf Eren Kaya. The authors also gratefully acknowledge the valuable support and guidance provided by Orkun Çınar during the development of this study. The authors would like to thank the Dokuz Eylül University Scientific Research Projects for their financial support.”

**References Correction**

The following three references have been added after Reference 39:

40. Bozkurt, E. Neotectonics of Turkey—A synthesis. *Geodin. Acta* **2001**, *14*, 3–30. https://doi.org/10.1080/09853111.2001.11432432.

41. Özkaymak, Ç.; Sözbilir, H.; Uzel, B. Neogene–Quaternary evolution of the Manisa Basin: Evidence for variation in the stress pattern of the İzmir-Balıkesir Transfer Zone, western Anatolia. *J. Geodyn.* **2013**, *65*, 117–135. https://doi.org/10.1016/j.jog.2012.06.004.

42. Emre, Ö.; Duman, T.Y.; Özalp, S.; Şaroğlu, F.; Olgun, Ş.; Elmacı, H.; Çan, T. Active fault database of Turkey. *Bull. Earthq. Eng.* **2018**, *16*, 3229–3275. https://doi.org/10.1007/s10518-016-0041-2.

With this correction, the order of some references has been adjusted accordingly.

The authors state that the scientific conclusions are unaffected. This correction was approved by the Academic Editor. The original publication has also been updated.
